# Dose rate correction for the novel 2D diode array MapCHECK 3

**DOI:** 10.1002/acm2.14471

**Published:** 2024-08-05

**Authors:** Mengyang Li, Yuan Tian, Linyi Shen, Guiyuan Li, Liang Zhao, Xinyuan Chen, Shouping Xu, Minghui Li, Peng Huang, Jianrong Dai

**Affiliations:** ^1^ Radiation Oncology Center, National Cancer Center/National Clinical Research Center for Cancer/Cancer Hospital Chinese Academy of Medical Sciences Beijing China; ^2^ Radiation Oncology Center, National Cancer Center/National Clinical Research Center for Cancer/Cancer Hospital (Langfang campus), Chinese Academy of Medical Sciences Langfang China

**Keywords:** correction, dose rate dependence, dose verification, MapCHECK 2, MapCHECK 3

## Abstract

**Purpose:**

To investigate the dose rate dependence of MapCHECK3 and its influence on measurement accuracy, as well as the effect of dose rate correction.

**Materials and methods:**

The average and instantaneous dose rate dependence of MapCHECK2 and MapCHECK3 were studied. The accuracy of measurements was investigated where the dose rate differed significantly between dose calibration of the MapCHECK and the measurement. Measurements investigated include: the central axis dose for different fields at different depths, off‐axis doses outside the field, and off‐axis doses along the wedge direction. Measurements using an ion chamber were taken as the reference. Exponential functions were fit to account for average and instantaneous dose rate dependence for MapCHECK3 and used for dose rate correction. The effect of the dose rate correction was studied by comparing the differences between the measurements for MapCHECK (with and without the correction) and the reference.

**Results:**

The maximum dose rate dependence of MapCHECK3 is greater than 2.5%. If the dose calibration factor derived from a 10 × 10 cm^2^ open field at 10 cm depth was used for measurements, the average differences in central diode dose were 0.8% ± 1.0% and 1.0% ± 0.8% for the studied field sizes and measurement depths, respectively. The introduction of wedge would not only induce −1.8% ± 1.3% difference in central diode dose, but also overestimate the effective wedge angle. After the instantaneous dose rate correction, above differences can be changed to 1.9% ± 8.1%, 0.2% ± 0.1%, and 0.0% ± 0.9%. The pass rate can be improved from 98.4% to 98.8%, 98.3%–100.0%, and 96.3%–100.0%, respectively.

**Conclusion:**

Compared with MapCHECK2 (SunPoint1 diodes), the more pronounced dose rate dependence of MapCHECK3 (SunPoint2 diodes) should be carefully considered. To ensure highly accurate measurement, it is suggested to perform the dose calibration at the same condition where measurement will be performed. Otherwise, the dose rate correction should be applied.

## 1 INTRODUCTION

Two‐dimensional (2D) detector arrays have become more widely used in clinics due to their convenience for dose verification (such as machine quality assurance [QA], and patient QA). Several 2D arrays are commercially available.^1^, ^2^, ^3^, ^4^, ^5^, ^6^, ^7^, ^8^, ^9^, ^10^, ^11^, ^12^, ^13^, ^14^, ^15^, ^16^, ^17^, ^18^, ^19^

Diode‐based arrays have smaller detector dimensions and therefore a narrower dose response function compared to ion chamber‐based 2D arrays. They have the ability to avoid the volume effect, which flattens the dose in regions with rapidly changing dose gradients.^19^

However, diodes are heavily dependent on the dose rate at the point of measurement as well as on the energy spectrum.^10^, ^20^ The dependence on the dose rate for the diode, especially for the n‐type diode,^21^ is always the biggest concern, as reported in many studies^20^, ^21^, ^22^, ^23^ on the diode based 2D array. The dose rate dependence can be further classified into average dose rate (ADR) dependence that can be explained by the traps theory and instantaneous dose rate (IDR or dose per pulse) dependence that can be explained by the recombination‐generation (R‐G) center theory.^24^, ^25^, ^26^

As a representative product of 2D diode array, MapCHECK was released by Sun Nuclear in 2002.^9^ In 2010, the second generation of MapCHECK, MapCHECK 2 (MC2), was used in our clinic for machine QA and collapsed‐angle IMRT QA. It equips with the same SunPoint 1 diodes (type 1) as MapCHECK but has an increased number (1527) of diodes and a larger measurement area (26 × 32 cm^2^).^14^ After array calibration and dose calibration at a certain depth followed by the instructions provided by the user manual, we use MapCHECK 2 for dose verification for different fields at different depths, and achieved satisfactory results. Recently, we have purchased the latest version of MapCHECK, MapCHECK 3 (MC3) for clinical application. It has the same number of diodes and measurement area as MC2, and features a new diode, the SunPoint 2 (type 4), which has a much smaller active area of 0.48 × 0.48 mm^2^ and significantly higher sensitivity of approximately 15 nC/Gy. The inherent buildup and backscatter have been slightly modified.

We found the pass rate decreased when switching measurement from MC2 to MC3. The large decrease in pass rate is particularly evident in measurements where the measured dose rate of the diode differs significantly between dose calibration and measurement due to the changes in field size, measurement depth, and other factors. It was similar with the findings of a previous study,^24^ which showed a large drop in pass rate due to the dose rate dependence of SunPoint 1 diodes. This encouraged us to perform a comparative study of the dose rate dependence for MC2 and MC3. The accuracy of the measurements where the measured dose rate of the diode differs significantly between dose calibration and measurement was investigated, such as the output (due to the change in field size), depth dose (due to the change in measurement depth), off‐axis dose outside the field (due to the block and attenuation of collimators), as well as the off‐axis profile along the wedge (due to the attenuation of wedges). The influence of the dose rate correction on the dose verification results was also reported.

## 2 MATERIALS AND METHODS

Measurements were performed with an Elekta Axesse LINAC (Elekta, Crawley, UK), which provided a 6MV x‐ray beam. The LINAC was carefully commissioned using a PTW BEAMScan watertank and appropriate ion chambers according to relevant standards.^27^ The percentage depth dose at 10 cm depth (PDD_10_) was 67.5% with 100 cm source‐to‐skin‐distance (SSD) and the absolute dose was calibrated as 100 MU deposited 1 Gy at the maximum dose depth (1.5 cm), according to the IAEA TRS 398 report.^28^

The surfaces of the MC2 and MC3 were perpendicular to the central axis (CAX) of the beam for all the measurements. SNC Patient software (version 8.4.1) was used to collect and save the data during MC2 and MC3 measurements. For the purpose of comparison, measurements with ion chamber (PTW 31021) calibrated to the IAEA TRS‐398 standard at the National Institute of Metrology, China, under the same condition were taken as the reference. The recombination effect of the ion chamber was investigated and found to be less than 0.3% for various dose rates.

For both MC2 and MC3, array calibrations were carried out by a stepwise calibration process designed by the manufacturer. Dose calibration was performed with a 10 × 10 cm^2^ open field and LINAC output dose rate of 309 MU/min under the standard conditions (listed in Table 1). There is a 0.05 cm difference in the total backscatter between MC2 and MC3 due to the difference in intrinsic backscatter between the two devices, as well as the limited availability of solid water slabs in our institute.

**TABLE 1:** Standard condition of measurement.

	MC2	MC3
Source‐to‐detector‐distance (SDD)	100 cm	100 cm
Intrinsic physical buildup	1.2 cm	1.2 cm
Intrinsic water equivalent buildup	2.0 cm	1.5 cm
Additional solid water buildup (30 × 30 cm^2^)	8.0 cm	8.5 cm
Total water equivalent buildup	10.0 cm	10.0 cm
Intrinsic physical backscatter	1.83 cm	1.8 cm
Intrinsic water equivalent backscatter	2.75 cm	2.3 cm
Additional solid water backscatter (30 × 30 cm^2^)	5.0 cm	5.5 cm
Total water equivalent backscatter	7.75 cm	7.8 cm
Dose	100 MU	100 MU

Both MC2 and MC3, as well as the ion chamber used in this study, were continuously connected to AC power and stored in the treatment room for at least 1 day to reach temperature equilibrium before calibration and measurements. All measurements were repeated four times.

### 2.1 Dose rate dependence

According to the previous studies,^24^, ^25^, ^26^ the dose rate dependence was further divided into ADR and IDR dependence, and the two types of dependence were measured and compared between MC2 and MC3 for the open field.

### 2.2 ADR dependence

The CAX doses of a 10 × 10 cm^2^ open field were measured by the PTW 31021 ion chamber under the standard conditions with different LINAC output dose rates ranging from 36 to 590 MU/min. The result with a LINAC output dose rate of 309 MU/min therein was used for dose calibration of MC2 and MC3 under the same measurement conditions. The central diode doses of MC2 and MC3 were then measured with different LINAC output dose rates. The dose readings were normalized to respective measurements with LINAC output dose rate of 309 MU/min. The dose rate dependencies were compared between MC2 (or MC3) and the ion chamber.

### 2.3 IDR dependence

IDR, or named dose‐per pulse (dpp), is another influence factor related to dose rate dependence that can affect the sensitivity of the diode. The IDR dependence was measured by the method proposed by Shi et al.^25^ The source to detector distance (SDD) was varied from 80 to 200 cm in steps of 10 cm while keeping the pulse repetition frequency constant at 309 MU/min. The field size (10 × 10 cm^2^) and total thickness (10 cm) of buildup were kept unchanged during the measurements.

### 2.4 CAX dose for different field size

For fields with different sizes from those (e.g., 10 × 10 cm^2^) used for calibration, both the phantom scatter factor, Sp, and the collimator scatter factor, Sc, would change. Hence, the measured dose rate of the CAX diode would vary with the size of the field, even if the average dose rate of LINAC remained constant. CAX doses of different field sizes ranging from 2 × 2 to 30 × 30 cm^2^ (2 × 2, 3 × 3, 5 × 5, 8 × 8, 10 × 10, 12 × 12, 15 × 15, 18 × 18, 20 × 20, 22 × 22, 24 × 24, 25 × 25, 28 × 28, 30 × 30 cm^2^) were measured by PTW 31021 ion chamber under the standard conditions. Central diode doses of all the fields were also measured using MC2 and MC3 and compared with those measured by ion chamber.

### 2.5 CAX dose at different depth

Variation in the measurement depth would change the attenuation of beam and cause the change in measured dose rate of CAX diode from calibration, even if the same field and average dose rate of LINAC were used. After dose calibration using a 10 × 10 cm^2^ open field at the depth of 10 cm, MC2 and MC3 were used to measure the 10 × 10 cm^2^ open field under the standard conditions (except for SDD = 100 cm) at different depths. The constant LINAC output dose rates of 309 MU/min were used for all these measurements and calibrations. The depth doses for a 10 × 10 cm^2^ open field under the same condition acquired by a PTW 31021 ion chamber in the solid water were taken as the reference to compare with the central diode doses of MC2 and MC3.

### 2.6 Off‐axis dose outside the field

The differences in off‐axis dose outside the field between the planned dose and the measured dose by 2D arrays can help to verify the transmission of the multi‐leaf collimator (MLC) or jaw in the beam model. However, due to the block and attenuation of collimators, the measured dose rate of diodes outside the field would vary from the diodes in the field and calibration. Doses for the points outside the field (with the off‐axis distance of 9 cm, OAP9, and 10 cm, OAP10) were extracted from the MC2 and MC3 measured files for a 10 × 10 cm^2^ open filed under the standard condition, and compared with the reference which measured by ion chamber in the solid water under the same condition.

### 2.7 Off‐axis dose along the wedge direction

The use of wedge would not only change the measured dose rate of CAX diode from calibration but also the measured dose rate of off‐axis diodes along the wedge direction. So, besides the measurements of the open field, we also compared the off‐axis dose along the wedge direction extracted from the MC2 and MC3 measurement file for 20 ×20 cm^2^ field with wedge 60° with those measured by the ion chamber under the same condition.

### 2.8 Correction

(1) $$CorrectedCount_{i}=\left(RawCount_{i}-bcg_{i}\cdot BeamOn\right)\cdot CF_{all}$$

Where, *CorrectedCount*_i_ is the cumulative corrected count for the diode i; *RawCount*_i_ is the cumulative raw count for the diode i; *bcg*_i_ is the background calibration factor for the diode i; *BeamOn* is the total time of beam delivery; *CF*_all_ represents the product of all calibration factors present in the file.

(2) $$DoseCount_{i}=DF\cdot CorrectedCount_{i}$$

where *DoseCount*_i_ is the cumulative dose for the diode i; *DF* is the dose calibration factor.

In the data file for MapCHECK 3, there are parameters such as beam‐on time, temperature calibration factor, pressure calibration factor, and array calibration factor, as well as data matrices including raw counts, corrected counts, background, and dose counts. The detail can be obtained from the user guide of the SNC Patient software. According to the Equations (1) and (2), raw counts matric can be corrected into dose matric. However, this correction method provided by SNC Patient software cannot correct the dose rate dependence of MapCHECK 3. Therefore, prior to the commencement of the aforementioned calculations, it is necessary to establish a fitting function to correct the influence on raw counts caused by dose rate dependence.

Due to the fact that with increasing dose rate the response converges to a maximum, an exponential saturation function^24^ was chosen to fit the relative diode response, *y*. The independent variables, *x*, were counts/50 ms and dose per pulse for ADR and IDR dependence, respectively. Counts/50 ms were calculated by averaging the plateau count/50 ms over the effective measuring time, while the doses per pulse were calculated by dividing the total doses with the total pulses.

(3) $$y=a\;e^{-\frac{x}{b}}+c.$$

where, *a*, *b*, and *c* are fitting parameters. Due to the lack of records of the number of updates and total pulses in the measurement file for MC2, the fit functions were studied only for MC3. Using the fit functions, the raw counts of each diode in the measurement of output and depth dose were corrected. The correction was applied to the total accumulated reading of each diode. The corrected raw counts were then used to calculate the corrected dose according to the user guide of MC3. The measured doses with and without correction were compared with the reference measured by ion chamber as well as the planned doses calculated by Monaco TPS using the SNC Patient software with more stringent criteria (2%/2 mm). The accuracy of beam model in Monaco TPS was carefully verified. For different sizes of fields, the point doses at different depths and positions calculated by the Monaco TPS were compared with those measured by the ion chamber, the maximum deviation was less than 1%.

## 3 RESULTS

### 3.1 Dose rate dependence

#### 3.1.1 ADR dependence

**FIGURE 1:**
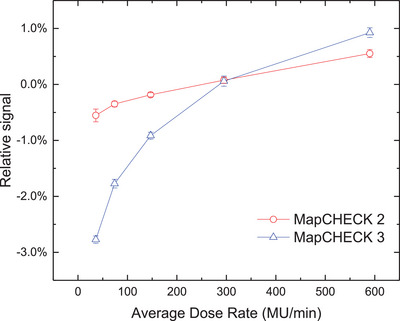
Differences in CAX dose between MC2 (MC3) and ion chamber with different ADRs.

Figure 1 illustrates the differences in CAX doses between the ion chamber and MC2 (or MC3) as a function of LINAC output dose rates. The CAX dose measured at a LINAC output dose rate of 309 MU/min was taken as a reference. As the LINAC output dose rate increases, the responses of these three detectors also increase. The ion chamber presents a good stability for LINAC output dose rates ranging from 36 to 590 MU/min, with the change in its sensitivity being less than 1%. The dose rate dependence of MC2 is slightly higher than the ion chamber, with the maximum sensitivity change being less than 1.0%. However, MC3 shows more pronounced average dose rate dependence, within the same range of LINAC output dose rate. The maximum sensitivity change is greater than 2.5%. It is worth noting that, if measurements were normalized by those obtained at the maximum LINAC output dose rate, the difference would be more pronounced.

### 3.2 IDR dependence

**FIGURE 2:**
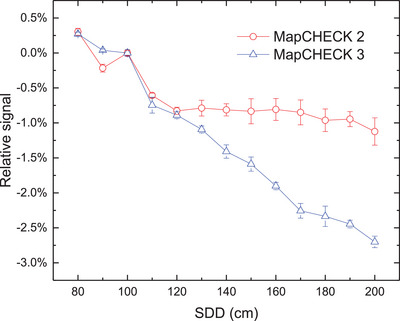
Differences in CAX dose between MC2 (MC3) and ion chamber with different SDD.

All CAX doses measured by ion chamber, MC2, and MC3 decrease with increasing SDD. As shown in Figure 2 when the SDD is less than the SDD used for dose calibration (SDD_calibration_), the central diode doses measured by MC2 and MC3 are both higher than that measured by the ion chamber. The differences decrease as the SDD increases. When the SDD is equal to SDD_calibration_, there is good agreement between all three measurements. However, when the SDD continues to increase and exceeds SDD_calibration_, the central diode doses measured by MC2 and MC3 become lower than those measured by the ion chamber. The differences gradually increase with the increasing SDD. Additionally, compared to MC2, MC3 shows a more pronounced IDR dependence, and the maximum difference compared with the ion chamber is lager than 2.5%.

### 3.3 CAX dose for different field size

**FIGURE 3:**
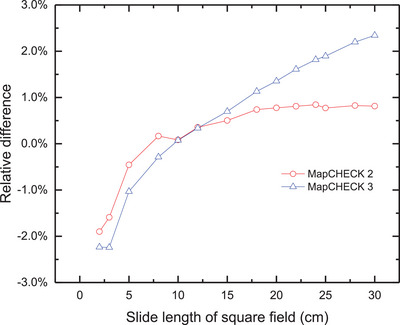
Difference in CAX dose between the measurements of MapCHECK (MC2 and MC3) and ion chamber for different sizes of fields.

Figure 3 shows the differences in CAX dose between ion chamber and MC2, as well as MC3, as a function of the field size under the standard conditions. When the field is larger than the reference field (10 × 10 cm^2^), the output factors measured by both MC2 and MC3 are larger than those measured by the ion chamber, and conversely for field sizes smaller than the reference field.

MC2 shows a good agreement with ion chamber with the maximum difference of less than −1.0% for field no smaller than 5 × 5 cm^2^. For 3 × 3 cm^2^ open field, the difference increases to −1.9%. We find that, MC3 is more sensitive to field size. The deviation between MC3 and the ion chamber is greater than 1.0% (absolute value) not only for fields smaller than 5 × 5 cm^2^ but also for fields larger than 18 × 18 cm^2^.

### 3.4 CAX dose at different depths

**FIGURE 4:**
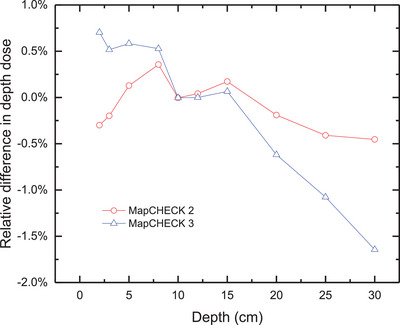
Difference in depth dose between MapCHECK (MC2 and MC3) and ion chamber.

Figure 4 shows the deviations in depth dose of MC2 and MC3 from the ion chamber. The depth doses measured by MC2 demonstrate good agreement with the ion chamber, with a maximum deviation of less than 0.5% for depths from 2 to 30 cm. However, there is a noticeable trend in the deviation of depth dose between MC3 and the ion chamber. For measurement depth smaller than the calibration depth (10 cm), the depth doses measured by MC3 are greater than those measured by the ion chamber. For depths greater than the calibration depth, the depth doses measured by MC3 are smaller than those measured by the ion chamber, and the deviation gradually increases with increasing measurement depth. For depths greater than 25 cm, the deviation exceeds −1.0%, and for depths greater than 30 cm, it exceeds −1.5%.

### 3.5 Off‐axis dose outside the field

The average differences in off‐axis dose between MC2 (MC3) and ion chamber are shown in Table 2. The mean deviation is −0.3% and −0.2% (−0.4% and −0.3%) for 9 and 10 cm off‐axis, respectively. It should be noted that the doses outside the field on both sides measured by MC2 and MC3 are lower than those measured by the ion chamber, and the deviations for MC2 is significantly (*p* = 3.4 × 10^−7^, paired *t*‐test) smaller than those for MC3. This cannot be explained by setup errors or steep dose gradients in the penumbra because these points are far from the penumbra.

**TABLE 2:** Differences in off‐axis dose outside the field between MapCHECK (MC2 and MC3) and ion chamber.

Point	Off‐axis distance	MC2 without correction	MC3 without correction	MC3 corrected by ADR correction	MC3 corrected by IDR correction
OAP9	9 cm	−0.3% ± 0.0%	−0.4% ± 0.0%	−0.2% ± 0.0%	−0.2% ± 0.0%
OAP10	10 cm	−0.2% ± 0.0%	−0.3% ± 0.0%	−0.2% ± 0.0%	−0.2% ± 0.0%

*Note*: IDR correction and ADR correction were applied to the measurements separately.

### 3.6 Off‐axis dose along the wedge direction

**FIGURE 5:**
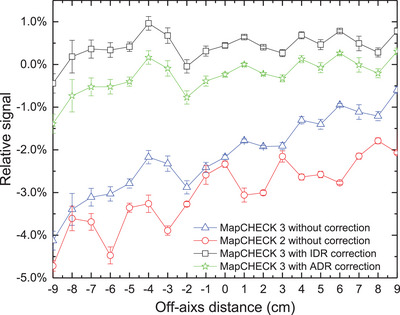
Differences in off‐axis doses along the wedge direction between MapCHECK (MC2 and MC3) and the ion chamber with and without dose rate correction. Note: The positive direction for the off‐axis distance is the toe of the wedge, and vice versa.

The relative differences in the off‐axis dose along the wedge direction between MC2 (or MC3) and the ion chamber are shown in Figure 5. The central diode doses decrease by −2.3% for MC2 (Figure 5, red circle), and −2.1% for MC3 (Figure 5, blue triangle), when compared to measurements taken by ion chamber. Additionally, the relative differences along the wedge direction show a decreasing trend from the thick side to the thin side of the wedge. This means that, if the dose calibration factor derived from an open 10 × 10 cm^2^ filed is used for MC2 and MC2 measurement of wedge field, not only the central diode dose (or wedge factor) would be underestimated, but also the effective wedge angle would be overestimated.

### 3.7 Correction

**FIGURE 6:**
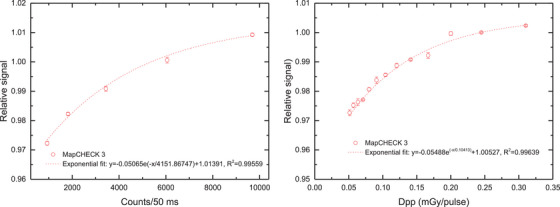
Fit function for ADR (left) and IDR (right) dependence for MC3.

Both ADR and IDR dependence were fitted well by exponential function with *R* ^2^ of 0.99559 and 0.99639, respectively (Figure 6). The two correction fit functions were applied to measurements separately.

As shown in Table 3, if ADR correction is applied, the average of absolute value of difference in CAX dose between MC3 and ion chamber decreases from 1.0% to 0.5% for depth dose, and a decrease of 0.04% for output was observed. The gamma pass rate with criteria of 2%/2 mm for depth dose is obviously improved from 98.3% to 100%, and a slightly increase of 0.03% for output was observed. After IDR correction, the pass rate further increases to 100.0% for measurements at different depths and 98.8% for different field sizes, respectively. The CAX dose deviation also further decreases to 0.2% for depth dose measurements, however, it increases to 1.9% with larger standard deviation for output dose measurements. This may result from the relative poor fit within dpp range (0.15–0.22) used for IDR correction (Figure 6, right).

**TABLE 3:** Comparison of gamma pass rate (average) and CAX dose deviation with and without dose rate correction.

	MC2 without correction	MC3 without correction	MC3 corrected by ADR correction	MC3 corrected by IDR correction
Measurements at different depths				
2%/2 mm	99.8%	98.3%	100.0%	100.0%
CAX dose deviation	0.6% ± 0.4%	1.0% ± 0.8%	0.5% ± 0.2%	0.2% ± 0.1%
Measurements for different filed size				
2%/2 mm	99.2%	98.4%	98.4%	98.8%
CAX dose deviation	0.6% ± 0.9%	0.8% ± 1.0%	0.8% ± 0.9%	1.9% ± 8.1%

The decrease of the average difference in off‐axis dose after ADR and IDR correction can also been observed in Table 2. For OAP9, the average difference is −0.4% before correction, and decreases to −0.2% and −0.2%, respectively after ADR and IDR correction. For OAP10, the same trend can be found.

ADR or IDR correction can significantly improve the accuracy of the measurement for wedge field (Figure 5). Not only does the difference in central diode dose decrease to less than 0.5%, but the difference in the off‐axis dose along the wedge direction can also be well corrected, thereby improving the measurement accuracy of the effective wedge angle.

Table 4 shows the pass rate and CAX dose deviation for different sizes of wedge fields with and without the dose rate correction. For field smaller than the reference field (e.g. 10 × 10 cm^2^), the CAX dose deviations are more significant compared to those for open field. However, for the field larger than the reference field, the deviation in CAX dose becomes smaller. The large CAX dose deviations (maximum to −5.4%) result in the lower pass rates (maximum to 82.0%) for small wedge fields. Both CAX dose deviations and pass rates of all the wedge fields are improved by dose rate correction. After ADR correction, the maximum CAX dose deviation and lowest pass rate are improved to −3.0% and 93.8%, respectively. Similarly, after IDR correction, the maximum CAX dose deviation and lowest pass rate are improved to −2.5% and 99.6%, respectively.

**TABLE 4:** Comparison of gamma pass rate (average) and CAX dose deviation with and without dose rate correction of MC3 under the wedge 60° field.

	MC3 without correction		MC3 corrected by ADR correction		MC3 corrected by IDR correction	
Wedge field size (cm^2^)	2%/2 mm	CAX dose deviation	2%/2 mm	CAX dose deviation	2%/2 mm	CAX dose deviation
2 × 2	93.8%	−5.4% ± 0.1%	98.5%	−3% ± 0.1%	100.0%	−2.5% ± 0.1%
3 × 3	84.4%	−4.2% ± 0.1%	93.8%	−2% ± 0.1%	100.0%	−1.3% ± 0.1%
5 × 5	82.0%	−2.1% ± 0.1%	100.0%	−0.1% ± 0.1%	100.0%	0.6% ± 0.1%
8 × 8	93.7%	−1.5% ± 0.1%	100.0%	0.2% ± 0.1%	100.0%	0.9% ± 0.1%
10 × 10	97.2%	−1.5% ± 0.1%	100.0%	0.2% ± 0.1%	100.0%	0.7% ± 0.1%
12 × 12	98.6%	−1.9% ± 0.1%	100.0%	−0.4% ± 0.1%	100.0%	0.1% ± 0.1%
15 × 15	99.0%	−1.6% ± 0.1%	100.0%	−0.3% ± 0.1%	100.0%	0.2% ± 0.1%
18 × 18	100.0%	−1.4% ± 0%	100.0%	−0.3% ± 0%	100.0%	0.2% ± 0%
20 × 20	100.0%	−1.3% ± 0.1%	100.0%	−0.2% ± 0.1%	100.0%	0.2% ± 0.1%
22 × 22	100.0%	−1% ± 0%	100.0%	0% ± 0%	100.0%	0.4% ± 0%
24 × 24	100.0%	−1.1% ± 0.1%	100.0%	−0.1% ± 0.1%	99.8%	0.2% ± 0.1%
25 × 25	100.0%	−1% ± 0.1%	100.0%	−0.1% ± 0.1%	99.6%	0.2% ± 0.1%
28 × 28	100.0%	−0.8% ± 0.1%	100.0%	0% ± 0.1%	100.0%	0.3% ± 0.1%
30 × 30	100.0%	−0.7% ± 0.1%	100.0%	0% ± 0.1%	100.0%	0.3% ± 0.1%
All fields	96.3% ± 5.9%	−1.8% ± 1.3%	99.5% ± 0.2%	0.5% ± 0.9%	100.0%	0.0% ± 0.9%

## 4 DISCUSSION

In this work, the dose rate dependence of MC2 equipped with SunPoint 1 and MC3 equipped with SunPoint 2 and their influence on measurements have been comprehensively investigated for a 6MV x‐ray beam.

The ADR dependence of MC3 in this paper seems larger than the statement of the manufacturer (±1.5% over the range 100−1400 MU/min). The reason may be that, first, the lowest dose rate measured in this study (36 MU/min) is lower than that stated by the manufacturer (100 MU/min). Second, the manufacturer does not provide the normalized dose rate in the statement of the user manual. If considering the same dose rate range (100–600 MU/min) and normalizing to the intermediate dose rate (∼300 MU/min), the dose rate dependence of MC3 measured in this work is generally consistent with the statement of the manufacturer.

MC2 has low dependence on the dose rate, as well as the field size and measurement depth, allowing it to be used to measure different field sizes at different depths after dose calibration at a certain depth with a certain field size. However, this experience is not appropriate for the MC3. Due to the more pronounced dose rate dependence, if the dose calibration factor of MC3 derived at a reference depth (e.g., 10 cm) with a reference field (e.g., 10 × 10 cm^2^) was used for measurements where the measured dose rates of diodes differed significantly from calibration, a nonnegligible error would be observed. For example, measurements for the fields (e.g., 2 × 2 cm^2^) smaller than the reference field would decrease the measured dose rates of CAX diodes due to the decreased collimator and phantom scatters, resulting in an underestimation (−2.2%) of the measured dose. Conversely, when the measured field size (e.g., 30 × 30 cm^2^) is larger than the reference field, the measured dose rate of the CAX diodes would increase, resulting in an overestimation (2.2%) of the measured dose. Similar situations can also occur in the measurements of depth dose, off‐axis dose outside the field, as well as the off‐axis dose along the wedge field. However, the reasons for the change in the measured dose rates of the diodes are build‐up attenuation, collimator block, and the introduction of the wedge, respectively.

Additionally, the effects on the measured dose rate caused by various factors will be superimposed or counteracted which may result in a more significant difference. The enlarged and reduced CAX dose deviations for small and large wedge fields, respectively compared to those for open fields, may be the comprehensive result of the effects of wedge introduction and field size on the measured dose rate of diodes. The introduction of wedge would always reduce the measured dose rate for all diodes, while the dose rate dependence caused by the size of the field depends on the size of the measured field relative to the reference field used for dose calibration. If the size of the measured wedge field is smaller than the reference field, the decrease in measured dose rate caused by the introduction of wedge and by the field size would result in an additive effect and a greater underestimation of the central diode dose. On the contrary, in the case when the size of the measured wedge field is larger than the reference field, the decrease in measured dose rate caused by the introduction of wedge would be partially counteracted by the increase in measured dose rate caused by the field size, resulting in a decrease in deviation of central diode dose.

To ensure accurate dose delivery, it is expected the dose distribution calculated by the TPS is in good agreement with the measurement as much as possible.^29^ It is crucial that the measurement must be accurate, otherwise, it will mislead the medical physicist into making an incorrect judgment. To account for the more pronounced dose rate dependence on the measurement accuracy, two methods can be used to reduce the differences in measured dose rates between calibration and measurements. The first one is to calibrate the dose as much as possible under the same condition (field size, measurement depth, average dose rate, with or without wedge) at which measurement will be performed. However, it is time‐consuming and not convenient for clinical use. The other one is to apply the dose rate correction for the measurements.

The SRS MapCHECK is equipped with the same SunPoint 2 diodes as MapCHECK 3.^30^ SNC patient software provides the average dose rate correction for SRS MapCHECK but no dose rate correction is available for MapCHECK 2 and MapCHECK 3. Admittedly, dose rate correction is more important for SRSMapCHECK because it is mainly used for dose verification of SRS plans which is characterized by larger dose rate change and smaller field. However, the nonnegligible error would be observed for MC 3 when the measured dose rates of diodes differ significantly from calibration as mentioned above.

After the dose rate correction proposed in this study, the differences between the measurements using MC3 and ion chamber can be reduced. The gamma pass rate could also be improved. The mean improvements over all measurements were not remarkable because for most of these measurements, the difference between the measured dose rate and the calibrated dose rate is not significant, and the error caused by dose rate dependence is very small. High pass rates have been achieved for these measurements even without the dose rate correction. However, for measurements with large differences in measured dose rate between calibration and measurement, dose rate correction can fully reflect its value. For example, if the dose was calibrated using a 10 × 10 cm^2^ open field at 10 cm depth, the pass rate for a 3 × 3 cm^2^ wedge field at 10 cm depth was only 84.4% with a CAX dose deviation of −4.2%. After IDR correction, the pass rate can be improved to 100% with a CAX dose deviation of −1.3%. The improvements are generally bigger for the IDR correction, compared to the ADR, which is consistent with the founding of earlier study.^24^

Our method for dose rate correction has good performance for the difference not only caused by a single factor but also by multiple factors, which means that the measurements for different conditions can be performed using a single dose calibration factor after appropriate dose rate correction. This would be more convenient for clinical use, even for the wedge field measurements.

The dose rate dependence of MapCHECK 3 is easier to detect and explore correction methods under simple field measurement (such as machine QA, TPS commissioning), but it does not directly indicate the impact of dose rate dependence and correction efforts on patient QA. In future research efforts, we will advance our investigation in this area.

## 5 CONCLUSION

In summary, MC3 equipped with SunPoint 2 diodes exhibits more pronounced dose rate dependence compared with MC2 equipped with SunPoint 1 diodes. The difference in field size, measurement depth, collimator blocks, and the introduction of wedge between calibration and measurement will result in the change in the measured dose rate of diodes (e.g., Volumetric Modulated Arc therapy). Therefore, for measurements where the measured dose rate may undergo significant changes and more accurate results are expected, special attention should be paid to the impact of the dose rate dependence of the diodes on measurement accuracy. It's better to perform dose calibration under the same condition (field size, measurement depth, average dose rate, with or without wedge) at which measurement will be performed to decrease the difference in measured dose rate between calibration and measurement as much as possible. Otherwise, the dose rate correction should be applied to the measurements.

## AUTHOR CONTRIBUTIONS

All authors have made substantial contributions to the work and development of this manuscript. All authors approved the manuscript. Mengyang Li, Yuan Tian, and Linyi Shen designed and performed experiments, and completed the manuscript. Guiyuan Li and Liang Zhao participated in measurements, scientific discussions, and manuscript writing. Xinyuan Chen, Minghui Li, and Peng Huang: participated in scientific discussions and helped with manuscript writing. Jianrong Dai: supervised the study and helped with writing of the manuscript.

## CONFLICT OF INTEREST STATEMENT

The authors declare no conflict of interest.
